# Supplementary material for: Are carbon emissions trading and green financial instruments synergistic? -Comprehensive quantitative research based on content analysis
Source: PLoS One. 2024 Mar 7;19(3):e0298601. doi: 10.1371/journal.pone.0298601 (PMC10919601; doi:10.1371/journal.pone.0298601)
Supplement: S1 Data — (ZIP) [file pone.0298601.s001.zip › Data/地级市Z_F工作报告.zip!/║╙▒▒/╨╧╠¿╩╨/└┤╫╘╣1⁄2╓┌║┼í╛┬φ┐╦╩2╛▌═°í┐_2017.docx]

各位代表：

　　现在，我代表邢台市人民政府，向大会报告工作，请予审议，并请政协委员和各位列席会议的同志提出意见。

　　一、过去四年的工作回顾

　　2013年以来，在以习近平同志为核心的党中央的坚强领导下，全市人民克服经济转型、环境治理、自然灾害等多重困难，坚持稳中求进工作总基调，贯彻省委、省政府和市委的决策部署，主动适应经济发展新常态，抢抓京津冀协同发展重大机遇，着力推进供给侧结构性改革，较好地完成了市十四届人大会议确定的任务目标，为全面建成小康社会奠定了坚实基础。

　　坚持以新理念引领新发展，综合实力不断增强。2016年，全市实现生产总值1954.8亿元，是2012年的1.3倍。全部财政收入194.4亿元，是2012年的1.1倍。公共财政预算收入111.5亿元，是2012年的1.3倍。固定资产投资2025.9亿元，是2012年的1.7倍。规模以上工业增加值632.4亿元，是2012年的1.1倍。社会消费品零售总额969.9亿元，是2012年的1.6倍。年末金融机构存贷款余额分别达到3335.3亿元、1995.9亿元，分别是2012年的1.6倍、1.7倍。

　　积极培育新动能，转型升级取得较大进展。累计压减炼铁271万吨、炼钢204万吨、煤炭235万吨、水泥196万吨、玻璃5970万重量箱、石膏73万吨。好孩子北方生产基地等57个重大产业项目竣工投产。装备制造业增加值占规模以上工业比重达到27.7%，成为第一支柱产业；规模以上高新技术产业增加值110亿元，占规模以上工业比重达到17.4%。粮食总产457.5万吨，新增威县国家级现代农业园区，农业产业化经营率69.1%，比2012年提高7.2个百分点。旅游业总收入178亿元，是2012年的2.7倍；进出口总额达到118.5亿元；2016年，第三产业对经济增长的贡献率达到56.7%，超过第一、第二产业之和。

　　抢抓京津冀协同发展重大机遇，新型城镇化步伐加快。城市总体规划获省政府批复，到2030年邢台市中心城区面积将达到170平方公里、人口达到170万。成功创建国家园林城市，省级园林县城全覆盖。邢东新区成为省级战略发展平台。市区集中供热率95%，比2012年提高13.5个百分点。常住人口城镇化率49.83%，比2012年提高6.98个百分点。威县、南和被列为国家新型城镇化综合试点县。石武高铁、邯（邢）黄铁路、京港澳高速改扩建、邢衡高速、邢汾高速竣工通行。邢台海关开关，“邢台号”高铁始发运行。建成南水北调配套水厂20座，西线引黄工程竣工。

　　以壮士断腕的决心治理污染，生态环境持续好转。强力推进大气污染治理，关停企业1289家，深度治理1816家，拆除燃煤锅炉2378台；全部取缔环市区小水泥、小石灰、小石子企业，退出关闭小煤矿42家；“气化邢台”、公交车出租车油改气、淘汰黄标车成效明显。与2013年相比，全市PM2.5、PM10平均浓度分别下降45.6%、51%，达标天数增加135天。全面推行“河长制”，18条河流启动治理，水体水质开始改善。深入实施太行山绿化攻坚，全市森林覆盖率提高4.2个百分点。

　　尽心尽力改善民生，人民群众得到更多实惠。民生支出累计1082.6亿元，占总支出的81%。城镇、农村居民人均可支配收入年均分别增长9.8%、11.1%。低保线、扶贫线“两线合一”，贫困发生率下降12个百分点，预计51万贫困人口稳定脱贫。累计新增城镇就业27.2万人，转移农村劳动力30.6万人次。社会保障标准提高，养老保险制度全覆盖，城乡居民医保整合走在全省前列。累计开工保障性住房和棚户区改造7.7万套，改造农村危房4.1万套。新增幼儿园595所，新改扩建中小学462所。公立医院综合改革深入推进，城市公立医院实现药品零差率销售。省级食品药品安全县全覆盖，安全生产、社会治安大局稳定，双拥共建、民族宗教、外事侨务、邮政通信、新闻广电、审计统计、人防地震、方志档案、老龄妇幼、残疾人事业、援疆援藏等工作都取得新成绩。

　　刚刚过去的2016年，在夺取抗击“7·19”特大暴雨洪灾全面胜利的同时，我市经济社会发展呈现出顽强的韧性和爆发力。全市实际利用外资、外商直接投资、限额企业消费品零售额、工业用电量四项指标增速全省第1，社会消费品零售总额、公共财政预算支出两项指标增速全省第2，全部财政收入、城镇居民人均可支配收入两项指标增速全省第3，规模以上工业增加值、固定资产投资、公共财政预算收入三项指标增速全省第4，生产总值、金融机构存款余额、农村居民人均可支配收入三项指标增速全省第5，主要经济指标增速均创近年来全省最好位次，实现了“十三五”良好开局。一年来，主要抓了以下工作：

　　（一）着力推进结构调整。实施“重大产业项目突破年”，250个省市重点项目完成投资406.4亿元。深入对接京津，开展合作项目170项，总投资1142亿元，5个共建产业园区加快建设。省级开发区完成整合，3家成为省级高新区。全面完成去产能任务。新增规模以上工业企业125家，中钢邢机获评全国首批制造业单项冠军示范企业。推动邢钢与中国钢研科技集团战略合作，谋划建设以钢为基材的新材料产业基地；推动建滔、旭阳与华能碳资产公司合作，谋划建设绿色煤化工基地；御捷、北汽隆威、开云、凯特、铭鹿等新能源汽车项目加快推进，低速电动车产能达到26.5万辆，从电极材料、电池、配件到整车，新能源汽车全产业链正在形成；组建信息产业集团，与北京航空航天大学开展战略合作，北航VR国重、北斗导航、无人机等新一代信息技术项目落户我市。以食品工业为代表的现代农业加速发展，光明集团全球单体最大的工厂化食用菌生产基地建成投产，总投资156亿元的13个合作项目加快推进，一个辐射带动邢台全域、投资超百亿、年销售收入近二百亿的现代产业园区已现雏形；今麦郎集团总投资16.2亿元的食品饮品智能化升级项目加快建设，将成为全国最大的方便食品生产基地；金沙河集团总投资6.5亿元的面粉及挂面生产线项目稳步实施，挂面年产量将超过110万吨，产销量全国第一；玉锋集团总投资17亿元的玉米深加工项目扎实推进，维生素B12生产能力全球第一；全市小麦、玉米加工量将分别达到640万吨、440万吨，相当于我市年产量的2.8倍和2倍。服务业稳步提升，引进域外金融机构3家，金融业增加值占生产总值比重达到4.4%，成为全市纳税第三的产业；京津冀首列直通中亚的“好望角”号国际货运班列顺利开通。

　　（二）着力加快新型城镇化建设。邢东新区获批国家级产城融合示范区，与华夏幸福、荣盛发展、中冶集团等达成战略合作，总投资55亿元的路网、管廊、学校、医院等基础设施项目开工。全市完成城建投资501.4亿元，17条道路工程加快推进，总投资25亿元的万达城市综合体项目启动。房地产违法建设整治全面展开，解决城建遗留问题56个，全市商品房销售面积353.8万平方米，同比增长36.6%。市区主要公共区域免费WiFi全覆盖；公共自行车一期4000辆投入使用；新增集中供热面积590万平方米。县城建设三年攻坚行动深入推进，完成城建重点工程250项，任县、威县通过国家园林县城初评。隆尧县莲子镇入选全国特色小镇，英谈、栾卸、神头、小河庄、西北留获评全国美丽宜居村庄。邢台机场、邢和铁路、邢台热电厂等重大工程加快推进。

　　（三）着力深化改革创新。积极推进“放管服”，在全省率先完成“三张清单”编制，建成市行政审批服务（公共资源交易）大厅，行政审批局县县全覆盖。积极推进商事制度改革，新增市场主体同比增长23.9%。“多规合一”、营改增、供销社、水资源税等改革有序推进。市属194家国有企业整合重组，顺德集团转型基本完成；建立了农业、林业、美丽乡村等一批投融资平台，设立了城市发展、产业引导等一批基金。总债务132亿元的邢衡、邢临两条高速公路上划，政府置换债大幅增加，降低了债务风险。实施省级以上科技创新项目95个，新增省级院士工作站9家、工程技术研究中心4家、产业技术研究院2家，新认定国家级众创空间2家。科技小巨人和科技型中小企业分别达到160家和4066家，增量、增幅全省第一。

　　（四）着力实施环境攻坚。散煤整治、露天矿山、焦化行业、道路车辆污染四大专项行动全面开展；“气化邢台”加快推进，淘汰燃煤锅炉830台，减少燃煤消耗100万吨；探索科技治污、第三方治理，推进玻璃产业超低排放；以“三个一批”为抓手，强化“10+3+6”冬防措施，大气污染治理超额完成省定任务。实行“河长制”，坚持“五必看、五必听”，4条监测出境河流达到省断面考核要求；坚持“一步到位、适度超前”，开展“城区污水处理能力提升三年攻坚行动”，市区污水处理二厂全面建设；地下水超采治理完成投资10.5亿元，压采地下水1.9亿立方米。全面启动土壤污染防治。100个停产整顿矿山全部关停。启动建设山区生态经济示范区24个，新增造林面积59.1万亩。

　　（五）着力增进群众福祉。坚持精准扶贫、精准脱贫，突出抓好产业扶贫、易地搬迁、政策兜底等工作，预计任县、南和、内丘、临西4个县和502个贫困村、9.86万贫困人口稳定脱贫。按照灾后重建“1+8+3”方案和“五步走”要求，如期完成各节点任务。在全省率先建立统一的居民基本医疗保险制度，企业职工养老保险缴费中断率下降5.8个百分点。开工棚户区改造安置住房1.6万套，基本建成1.9万套。成功推出新版河北梆子《吕玉兰》、话剧《李保国》等一批文艺精品，博物馆、示范性综合实践基地、市直第二幼儿园、人民医院新院区和市三院、市五院改扩建扎实推进，全面两孩政策顺利实施。成功举办首届国际公路自行车大赛，14个国家和地区的200多名运动员和3000多名爱好者参赛，展示了大美邢台的风采！修订规委会、土委会议事规则，建立政府工作微信群，市本级取消下放行政许可32项，坚持市民代表列席政府常务会，权力运行更加规范透明。扎实开展“两学一做”学习教育，坚决纠正“四风”，查处违反中央八项规定精神典型问题393起，党政纪处分337人。自觉接受人大法律监督、工作监督和政协民主监督，办理人大议案建议369件、政协提案717件。

　　各位代表！这些成绩的取得，是党中央国务院、省委省政府和中共邢台市委正确领导的结果，是人大、政协和社会各界监督支持的结果，是全市人民团结拼搏的结果。在此，我代表市政府，向全市人民，向人大代表、政协委员、各民主党派、工商联、无党派人士和人民团体，向驻市部队、武警官兵、政法干警和驻市各单位、各企业，向关心支持邢台的各位老领导、老同志、海内外朋友，致以崇高的敬意和衷心的感谢!

　　在肯定成绩的同时，我们也清醒地认识到发展中存在的突出矛盾和问题。经济发展质量效益不高，科技创新能力不强，实体经济发展困难，传统产业占比较大，新动能尚未形成支撑；城镇化水平滞后，城市功能不全，基础设施欠账很多；环境污染形势依然严峻，大气、水、土壤治理任务艰巨；城乡居民收入不高，公共服务水平较低，教育、医疗、养老、就业等方面与人民群众的期盼还有较大差距，一些重大安全事故令人痛心；政府职能转变还不到位，“低标准、过得去、穷对付”的现象不同程度存在，一些部门和人员担当意识、服务意识不强，营商环境亟待改善，消极腐败现象仍有发生。去年《政府工作报告》确定的211项重点工作，有27项没有完成。对于这些问题，我们一定认真加以解决。

　　各位代表，在过去几年的工作实践中，我们深切体会到，推动邢台改革发展，必须坚持党的领导，坚决拥护和捍卫以习近平同志为核心的党中央，自觉增强政治意识、大局意识、核心意识、看齐意识，确保中央和省市委决策部署落到实处。必须践行新发展理念，以创新的思维、赶超的意识、进取的姿态，把新发展理念落实到经济社会发展各个领域、各个环节，坚定不移走好加快转型、绿色发展、跨越提升的新路。必须推进改革开放，在改革创新中破解难题，在扩大开放中拓展空间，在对标先进中补齐短板。必须做到实干担当，提振干事创业精气神，夙兴夜寐、激情工作，以马上就办、办就办好的优良作风推动邢台又好又快发展。

　　二、未来五年的工作安排

　　未来五年，是邢台转型发展、走好新路的关键时期。全球经济深度调整，我国经济发展进入新常态，京津冀协同发展步入快车道，党中央、国务院决定设立雄安新区，我省全面启动产业转型升级试验区建设，这些都为我们调整结构、开放合作、引资引智提供了重大发展机遇。市第九次党代会规划了未来五年发展蓝图，邢台已站在一个新的历史起点，推动产业升级、动能转换有基础，推动生态优先、绿色发展有空间，推动改革创新、扩大开放有条件，建设经济强市、美丽邢台其时已至，其势已成。

　　今后五年政府工作的指导思想是：全面贯彻党的十八大和十八届三中、四中、五中、六中全会和省、市第九次党代会精神，深入学习贯彻习近平总书记系列重要讲话精神和治国理政新理念新思想新战略，统筹推进“五位一体”总体布局，协调推进“四个全面”战略布局，以新发展理念为指导，以供给侧结构性改革为主线，适应把握引领经济发展新常态，坚持稳中求进工作总基调，围绕“一个建成”目标和“五个方面新突破”要求，走新路、有作为、创亮点、守底线，坚定不移推进协同发展，坚定不移推进改革创新，坚定不移推进转型升级，坚定不移推进环境治理，坚定不移推进脱贫攻坚，全面建成小康社会，加快建设经济强市、美丽邢台。

　　未来五年，将努力形成经济增长新动能。深入实施创新驱动和“中国制造2025”发展战略，调结构、提层级，推动产业向中高端迈进。加快钢铁、煤炭、玻璃等传统产业转型升级，大力发展先进装备制造、新能源新材料、汽车及新能源汽车、食品医药产业，培育壮大生命健康、电子信息、节能环保产业，打造一批战略性新兴产业发展集聚区和特色产业集群，高新技术产业增加值占规上工业比重达到26%以上。深化农业供给侧结构性改革，以提高农业供给质量为主攻方向，以壮大食品工业为突破点，全面提升农产品标准化生产、品牌创建、质量安全水平，优化品种布局，深化农企对接，促进一二三产跨界融合，推动农业大市向农业强市转变。加快服务业模式创新、多维拓展，开展“互联网+流通”行动计划，着力建设冀中南物流枢纽城市，服务业增加值占生产总值比重达到45.5%以上；完善“快进慢游”交通网络，推动旅游全域化发展，旅游业收入实现翻番，建设中国北方重要的生态旅游名城。发挥科技创新引领作用，强化企业创新主体地位，培育壮大高新技术企业和科技型中小企业，推动知识密集型产业快速成长，每万人拥有专利达到5.8件以上。突出发挥邢台职业技术教育优势，强化办学质量和特色，深化产教融合、校企合作，努力把邢台建设成为全国重要的高技能人才培养基地。

　　未来五年，将全面加快新型城镇化进程。着眼建设京津冀新型城镇化与城乡统筹示范区，积极融入中原经济区城市群，大力推进以人为核心的新型城镇化，常住人口城镇化率达到62%左右。聚焦邢东新区战略平台，对接京津产业转移和科技创新资源，重点建设高铁中央商务区、上东综合商贸区和顺水河生态区，以产兴城、以城促产，打造现代化新城区、新中心、新增长极。以“六城同创”为引领，推动中心城区扩容提质，积极稳妥推进行政区划调整，抓好龙岗、七里河、开元顺德等大片区开发建设，加快市区排水防涝系统和城市水系等基础设施建设，完善城市功能，提升城市品质。统筹推进县城发展，支持宁晋、威县、清河建设次中心城市；培育发展特色小城镇，加快建设美丽乡村。立足京津冀交通一体化发展，完善交通体系规划，推进重大交通基础设施建设，形成内畅外联、互联互通的大交通格局。

　　未来五年，将全面打造生态宜居之地。始终坚持“绿水青山就是金山银山”，守牢生态、发展和民生底线。坚决打好蓝天保卫战，坚持标本兼治、主攻治本，突出结构减排，强力实施大气污染综合治理“四个攻坚战”，推广第三方治理模式，持续改善空气质量，PM2.5平均浓度降到60微克/立方米以下。全面落实“河长制”，“水、滩、岸、路、绿”综合施治，让全市的河流成为生命之河、美丽之源。用好南水北调引江水，“节、引、蓄、调、管”多措并举，全面治理地下水超采。完善生态功能分区，强化水源地保护与管理。深入开展“净土行动”，加强农业面源污染和土壤污染防治，促进土壤资源永续利用。着力建设国家公园、生态林场、骨干生态防护林体系和绿美廊道，协同推进全域生态化和生态产业化，全市森林覆盖率达到33%以上，让“绿满邢襄、水润古城”成为邢台生态底色。

　　未来五年，将全面构建全方位改革开放格局。聚焦重点领域，把握关键环节，深化供给侧结构性改革、行政体制改革、国有企业改革、商事制度改革、农业农村改革、金融财税和投融资体制改革，努力趟出一条具有邢台特点的改革路径。扩大开放，精准对接，务实融入京津冀协同发展。夯实基础、培育特色，抓好承接平台建设，引进京津科技、教育、医疗、文化、总部基地。进一步理顺管理体制，深化人事、薪酬等制度改革，推动开发区完善功能，增强活力，引进龙头企业、知名品牌和重大项目，推动招商引资和承接产业转移向高端化迈进。抢抓“一带一路”等重大战略机遇，吸引更多的战略投资者，鼓励优势企业、产业走出国门，拓展发展空间。推进简政放权、放管结合、优化服务，构建“亲”“清”新型政商关系，真正理解、尊重企业家，激发和保护企业家精神，营造创新创业的浓厚氛围。

　　未来五年，将全面提高人民群众的获得感和幸福指数。落实“六个精准”“五个一批”要求，实现现行标准下农村贫困人口稳定脱贫，贫困村如期出列，贫困县全部摘帽，确保小康路上一个都不掉队。实施更加积极的创业就业政策，努力创造更多的就业岗位。以改革创新为引领，理顺教育管理体制，支持社会力量办学，扩大优质教育覆盖面，促进教育均衡发展；大力开展文艺精品创作，加强历史文化资源挖掘利用，积极做好文物保护，促进文化产业深度融合；实施健康邢台工程，推进“互联网+健康医疗”，发展中医药事业，加强基层医疗机构建设，提升公共卫生服务水平。健全以社会保险、社会救助、社会福利为基础，覆盖城乡的社会保障体系。发展老龄、慈善、残疾人事业，保障妇女儿童权益，关爱留守儿童。健全防灾减灾体系，提升防灾减灾水平。加强社会治理，建设法治邢台，完善食品药品长效监管机制，守牢安全生产底线，防范金融风险，始终保持对各类违法犯罪的高压态势，守护好“餐桌子、钱袋子、车轮子”，让邢台的百姓过得更有保障、更加幸福、更有尊严。

　　三、2017年的具体任务

　　2017年经济社会发展主要预期目标是:生产总值增长7.5%左右，规模以上工业增加值增长6%，公共财政预算收入增长7.5%，固定资产投资增长9%，社会消费品零售总额增长10%，出口总值增长1.5%，实际利用外资增长7%以上，城镇、农村居民人均可支配收入均增长8%，PM2.5平均浓度下降13.8%，化学需氧量、二氧化硫、氨氮、氮氧化物排放削减量完成省下达任务，每万元生产总值能耗下降4.1%，地表水水质优良比例达到41.9%以上。重点抓好八方面工作：

　　（一）扎实推进“三去一降一补”。去产能。按照省委、省政府要求，年内压减炼钢60万吨、煤炭423万吨、焦炭60万吨、玻璃260万重量箱，关停邢东热电厂、矸石热电厂，退出全部石膏产能。综合运用市场化法治化手段，有效处置僵尸企业。落实“五个一批”、援企稳岗等政策措施，确保职工可安置、生活有保障。严格排查核验，确保真去真退不反弹。去库存。坚持“房子是用来住的，不是用来炒的”定位，分类施策、因地调控，去化周期保持在合理区间，促进房地产市场健康发展。加大棚改项目支持力度，用好国开行、农发行等国家政策性资金，提高货币化安置率。去杠杆。规范政府举债行为，有效化解政府性债务风险，分类推进融资平台市场化转型。促进企业盘活存量资产，推进资产证券化，加大股权融资力度。降成本。落实减税降费政策，降低企业交易成本，支持实体经济发展。完善重点企业服务联席会议制度，一事一议破解发展难题。加大亏困企业帮扶力度，强化信息对接、金融支持、要素保障等服务，推动企业复产、达产。补短板。以回应群众期盼为着力点，加快补齐教育、文化、科技、医疗、体育等方面的短板。

　　（二）加快培育新的发展动能。以产业结构的调整优化，提升发展质量和效益。做大做强邢台制造。以装备制造为重点，开展新一轮技术改造和百项技改工程，推动“互联网+”和“两化”深度融合，新增规模以上工业企业90家。发挥全国玻璃期货基准价区作用，提升沙河玻璃产业层级和市场影响力。支持御捷、北汽隆威等项目建设，打造新能源汽车生产基地；全力抓好“两汽一拖”的二次创业，再圆邢台“汽车梦”。发展壮大宁晋电缆、临西轴承、平乡自行车等县域特色产业，建设一批超百亿元的特色产业集群。做强新能源产业，加快超威石墨烯蓄电池项目建设，晶澳年产1GW高效太阳能电池项目建成投产。抓好北航VR国重、北斗导航、无人机项目，培育以大智移云为主的新一代信息产业。弘扬工匠精神，强化品牌建设，新增驰名商标、著名商标30件以上，争创省级名牌产品46项。推进农业供给侧结构性改革。稳粮、优经、扩饲，粮食产量稳定在450万吨左右，创建市级以上畜禽养殖标准化示范场20个，调优蔬菜3万亩，压减棉花28万亩，扩大优质饲草4.7万亩。抓好今麦郎扩能升级、玉锋玉米深加工、光明玉兰农场等项目，支持金沙河、绿岭、富岗等龙头企业做大做强，各县市至少新增1个超亿元农业产业化项目，全市新增省级以上现代农业园区3家，农业产业化经营率提高1个百分点。持续深化农业农村改革，基本完成土地承包经营权确权登记，推广供销社新农协工作经验。支持嘉年华、红石沟等项目发展集循环农业、创意农业、农事体验于一体的田园综合体，壮大农村电商、农事节庆、乡村旅游等农业新业态。提升现代服务业水平。力争新引进2家以上域外金融机构，新增挂牌上市企业15家以上，加快电子商务发展，促进中亚国际货运班列常态化运行。抓好省级全域旅游示范县创建，重点打造大峡谷、前南峪、岐山湖、扁鹊庙、红石沟五大旅游片区，加快景区道路互联互通及旅游基础设施建设，举办好全市首届旅游业发展大会，旅游业总收入同比增长20%以上，突破200亿元。

　　（三）着力扩大有效投资。以项目促转型，以项目增财力，以项目促发展。坚持月通报、季调度、半年观摩和重大项目领导分包等制度，完善项目第三方机构核查、考评激励和动态调整机制，对重点项目优先配置土地资源、优先享受政策和资金支持。抓好石药集团宁晋生物科技产业园等总投资588亿元的27个省重点项目，年度完成投资75亿元；实施市重点项目260个，年度完成投资420亿元。清河新能风电等50个前期项目争取尽快开工，邢台一拖现代农装机械制造等45个项目实现竣工投产。深化邢东新区与华夏幸福、荣盛发展的合作，力促华夏幸福产业港、产业新城等项目落地。以列入全国PPP创新工作重点城市为契机，打好“两降两扩”组合拳，激发民间资本投资活力。完善开发区基础配套，抓好“新九通一平”建设。着力培育市开发区、沙河市、宁晋县3家千亿级园区，加快建设一批“园中园”、中外产业园。全方位对接京津，加强与大企业大集团的合作，年内力争引进2个100亿元项目、10个50亿元项目、50个10亿元项目及10个1亿美元以上的外资项目。

　　（四）全面深化改革创新。坚持创新驱动、改革推动，有效增强转型发展内生动力。深化行政体制改革。聚焦项目建设和企业登记注册等重点领域，继续下放一批市本级审批事项。组建市行政审批局，加快推进“互联网+政务服务”。用好“多规合一”规划成果，完善“双随机、一公开”监管制度。加强诚信体系建设，严肃查处破坏发展环境和市场经济秩序的违纪违法行为。做强做优国有企业。加大经营性国有资产集中统一监管力度，完善“1+14”政策体系，完成董事会授权、混合所有制、职业经理人制度以及国有资本投资运营“四项试点”工作，全面推进“三供一业”分离移交。加快市属国有企业负责人薪酬制度改革，推进顺德集团、水业集团、路桥集团、燃气热力集团、环卫集团等企事业单位的改革。创新财政金融体制。深化国地税征管体制和绩效预算管理改革，实施财政资金金融化改革，完善市级产业引导股权投资“母基金”，组建5支“子基金”，总规模力争达到50亿元；设立“蓝天”基金、过桥基金。优化金融生态环境，引导金融机构创新产品，发展证券、担保、融资租赁等金融业态，提升服务“三农”、实体经济和小微企业的水平。深化行政事业单位国有资产管理改革，政府公物仓建成投用。大力推进科技创新。推动市知识产权交易平台、市科技综合服务平台建设，新增省级院士工作站2家、产业技术研究院1家，新增科技型中小企业1000家、高新技术企业50家、科技小巨人企业40家、市级以上众创空间5家。推进人才评价机制改革，评选首届科学技术突出贡献奖。

　　（五）努力提升城乡建设水平。坚持“做优老城、做大新城、做强县城、做美村镇”，进一步改善人居环境，常住人口城镇化率提高2.4个百分点左右。政府投资160亿元以上，带动总投资1500亿元以上，实施九大类、225项重点城建工程。构建大交通格局。启动市区轨道交通工程，加快邢和铁路建设，用两年左右时间完成高铁综合交通枢纽、环城公路、邢台机场、太行山高速、邢石大道建设和邢清线、308线改造。完善城市功能。抓好博物馆、规划馆、国际会展中心、金融中心、工会大厦等重大城建工程，打造城市地标。贯通建设大街东延、新华路北延、兴达路南延、太行路北延、学院路等12条断头路，建设莲池大街立交桥，在繁华路段建设过街天桥或地下通道。加快推进泉北大街、振兴一路、心河路地下综合管廊工程，抓好钢铁路、中兴大街等86条道路改造提升。新增集中供热面积420万平方米。打造城市园林水系。加快建设14平方公里的中央生态公园，提升开元路、守敬路、新兴大街等10条街道绿化彩化水平，新建12个街头游园，加快构建市区及周边“一区、两道、四带、八园”绿化格局，新增相当于120个达活泉公园的绿化面积。全市新增造林面积50万亩以上，森林覆盖率超过30%。对七里河、牛尾河、小黄河、茶棚沟、围寨河进行治理，因地制宜扩大水面，打造亲水工程、靓丽水景，努力实现“绿满邢襄、水润古城”。提升城市品质。加快智慧城市建设，实现数字化城管平台全覆盖。实施亮净工程，开展市区环境综合整治百日攻坚，加强出市口整治，拆除违章违建，消除“城市疤痕”。桑德垃圾焚烧厂正式运行，新建大型生活垃圾压缩转运站，完成市区90座厕所改造提升。推广“一网三会”模式，提升社区物业管理服务水平。扩大公共自行车覆盖面。加快县乡村建设。深入推进县城建设攻坚行动，年度投资160亿元以上，全面实施“一补三化”，加快清河、威县海绵城市建设，各县市至少新建或改造提升1个特色风貌街区、2个以上精品建筑、1座四星级以上精品公园，县级主城区实现公共区域免费WiFi全覆盖。依托产业、人文和生态优势，加大24个重点镇培育力度，建设一批特色小镇；抓好561个省重点村建设，加快推进“三区同建”，全面开展“两改一清一拆”，建设美丽家园，培育良好村风。

　　（六）持续改善生态环境。打好蓝天保卫战。坚持科学治霾、协同治霾、铁腕治霾，全面实施“1+1+19”大气污染防治专项方案。大力开展农村散煤治理，完成煤改气（煤改电）21.8万户，市区实现“无煤化”；取缔10蒸吨及以下燃煤锅炉2357台，净削减燃煤总量35万吨。加大“小散乱污”企业整治力度。加强面源污染管控，强化机动车管理，全面供应国六标准汽柴油，增加新能源公交车；提升精细化管理水平，市区道路机械化清扫率达到三分之二以上；加强矿山企业扬尘治理，完成56处责任主体灭失露天矿山迹地修复绿化。下大力治理水污染。落实国家“水十条”和省“水五十条”，继续实施城区污水处理三年攻坚，2018年底市区基本实现雨污分流、污水处理达标、中水回用“三个百分之百”。完善“河长制”，河流退污还清取得实质进展。全面落实地下水压采任务，做好引黄调水工作，消纳江水1亿立方米以上。完成乡镇以上集中式饮用水水源地保护区划定，实现朱庄水库备用水源地一级保护区防护隔离，生活饮用水监测全覆盖。加强土壤污染防治。落实“土十条”要求，实施“净土行动”，建立分类分级管理制度，严控工业污染物排放和农业面源污染，扎实做好受污染农用地集中治理。做好第二次全省污染源普查准备工作，划定生态红线。持续开展利剑斩污行动，严格落实“三个一批”，始终保持环境执法高压态势。

　　（七）坚决打好脱贫攻坚战。聚焦聚力脱贫攻坚，下足“绣花”功夫，确保3个国定贫困县脱贫摘帽，6.5万贫困人口稳定脱贫。大力发展扶贫产业。调动贫困群众内在积极性，完善推广“资金入股”“资产收益”等产业扶贫新模式，做大做强特色种植、特色养殖、家庭手工、农村电商、光伏发电和旅游服务六大扶贫业态，实施好180个扶贫开发项目，带动4万贫困人口稳定脱贫。实施贫困村提升工程。加快首批易地扶贫搬迁工程进度，6月底达到入住条件；启动内丘、临城18个村7236人易地扶贫搬迁。按照产业园区和移民社区“两区共建”要求，真正实现搬得出、稳得住、能致富。坚持生态扶贫“四位一体”，抓好176个村硬化、亮化、绿化、文化广场等基础设施建设。发挥政策集成作用。提高农村低保标准和补差标准，落实好“三免一助”“雨露计划”和农村义务教育学生营养改善计划，着力解决贫困群众因病、因学、因灾、因房返贫问题。用足用好支持贫困地区IPO政策。落实脱贫攻坚责任。发挥考核“指挥棒”作用，砸实贫困县党委、政府主体责任，坚决落实不脱贫不脱钩要求。做好中直、省直单位及部队对口帮扶对接服务工作，深化工作队驻村帮扶，扎实推进社会帮扶。盯住资金使用、项目实施、政策落实等关键环节，加强督导，强化考核，确保扶贫工作务实、脱贫过程扎实、脱贫结果真实。

　　（八）倾力改善社会民生。牢固树立以人民为中心的发展理念，努力让人民群众生活得更美好。全力推进灾后重建。按照“1+8+3”方案要求完成各项节点任务，重点抓好顺水河整治、大贤桥等项目，入汛前完成各项防汛设施修复重建，年底前确保灾区群众生产生活条件达到或超过灾前水平。青山水库、白马河治理、八里庙抽水蓄能电站、大陆泽宁晋泊蓄滞洪区安全建设、平原水库和湿地等重大水利工程取得实质进展。全面提升气象水文监测预报预警能力。突出抓好社会保障。实施社保扩面提标工程，推进机关事业单位养老保险制度改革，力争企业职工养老保险中断缴费率下降5个百分点；探索医疗保险异地报销直接结算，加快医疗保险县市区并网进度。更加关注复转军人安置、大中专毕业生就业，城镇新增就业6.2万人，城镇登记失业率控制在4.5%以内。发展教卫文体事业。实施市区学校建设三年攻坚行动，市区新改扩建5所幼儿园、12所小学、7所初中、2所高中。探索学校建设新模式、校长管理新体制、教师补充新机制，实现教育城域网全覆盖。提升职业教育质量，抓好3所精品中职、5所品牌中职、2所特色中职学校建设。支持邢台医专专升本和邢台学院硕士立项建设。坚持三医联动，全面深化公立医院改革，加快建设市人民医院新院区，谋划眼健康基地。实施文艺工作者下基层、24小时自助图书馆等文化惠民措施。加强国家高水平体育后备人才基地建设，筹办好第二届国际公路自行车大赛。维护社会和谐稳定。开展安全生产百日攻坚行动，坚决遏制重特大事故发生。谋划建设市应急指挥中心，加强各项应急演练，提升预判预警和突发事件应急处置能力。严防严管严控食品药品安全风险。加大防范和打击非法集资力度，确保不发生系统性区域性金融风险。推进立体化、信息化社会治安防控体系建设，依法严惩黑恶势力和违法犯罪行为。创新和加强社会治理，扎实做好信访维稳工作，用心用情用力解决好群众诉求。

　　加强国防教育和国防后备力量建设，支持国防和军队改革，着力解决随军家属就业和子女入学问题。推动民族宗教、外事侨务、邮政通信、新闻广电、审计统计、人防地震、方志档案、老龄妇幼、残疾人事业、援疆援藏等工作取得新进步。

　　四、全面加强政府自身建设为人民服务，对人民负责，受人民监督，让人民满意，是政府必须坚守的信念；完成目标任务，提升落实能力和施政水平，是政府自身建设的永恒主题。

　　（一）坚定信念，提升素质。增强“四个意识”，始终在思想上政治上行动上同以习近平同志为核心的党中央保持高度一致，确保政令畅通、令行禁止。深入学习习近平总书记系列重要讲话精神和治国理政新理念新思想新战略，推进“两学一做”学习教育常态化制度化。提升主动适应新常态的能力水平，克服本领恐慌。探索机关内部轮岗机制，强化教育培训和实践锻炼，多向群众学习，多到基层走访，多听群众呼声，提升公务员的素质能力，增强政府工作的针对性、实效性。

　　（二）依法行政，履职尽责。深入贯彻全面依法治国要求，强力推进依法治市，尊崇法治、敬畏法律、依法办事。自觉接受人大的法律监督和工作监督，政协的民主监督，新闻舆论、网络媒体和社会公众监督，认真落实人大决议，办理好人大议案建议和政协提案。广泛听取民主党派、工商联、无党派人士和各人民团体意见。健全公众参与、专家论证、风险评估、合法性审查制度，确保依法、科学、民主决策。

　　（三）转变作风，实干实政。巩固“走新路、有作为、创亮点、守底线”解放思想大讨论成果，事争一流、唯旗是夺，对标先进、追求极致，守住底线、真抓实干。持续深化机关作风整顿，着力解决怠政懒政、不作为、慢作为、乱作为行为。坚持工作“晾晒”，围绕任务目标，年初建账、年中查账、年底算账，摆问题、找差距、促落实。坚持约束和激励并举，健全激励机制和容错纠错机制，为干事者鼓劲，为担当者担当。

　　（四）廉洁自律，干净干事。坚持纪严于法、纪在法前，严明政治纪律，恪守政治规矩，履行主体责任和“一岗双责”，落实党风廉政建设责任制。认真贯彻《准则》和《条例》，严格落实全面从严治党要求，保持政治定力，站稳人民立场，严格廉洁自律，不为任何利益群体所围猎。强化减权限权、公开透明，严查权力寻租，加强政府投资类项目监管，推动监察和审计全覆盖，让政府权力始终在阳光下运行。

　　各位代表，市第九次党代会开启了建设经济强市、美丽邢台的新征程。让我们紧密地团结在以习近平同志为核心的党中央周围，在中共邢台市委的正确领导下，不忘初心，继续前进，撸起袖子加油干，以优异成绩迎接党的十九大胜利召开！
